# Metformin alleviates lung-endothelial hyperpermeability by regulating cofilin-1/PP2AC pathway

**DOI:** 10.3389/fphar.2023.1211460

**Published:** 2023-06-08

**Authors:** M. Rizwan Siddiqui, Narsa M. Reddy, Hafeez M. Faridi, Mohd Shahid, Thomas P. Shanley

**Affiliations:** ^1^ Department of Pediatrics, Ann & Robert H. Lurie Children’s Hospital of Chicago, Stanley Manne Children’s Research Institute, Northwestern University Feinberg School of Medicine, Chicago, IL, United States; ^2^ Drug Discovery Center, Department of Internal Medicine, Rush University Medical Center, Chicago, IL, United States; ^3^ Department of Pharmaceutical Sciences, Rosalind Franklin University of Medicine and Science, North Chicago, IL, United States

**Keywords:** metformin, PP2A, cofilin-1, vascular endothelial cells, acute lung injury

## Abstract

**Background:** Microvascular endothelial hyperpermeability is an earliest pathological hallmark in Acute Lung Injury (ALI), which progressively leads to Acute Respiratory Distress Syndrome (ARDS). Recently, vascular protective and anti-inflammatory effect of metformin, irrespective of glycemic control, has garnered significant interest. However, the underlying molecular mechanism(s) of metformin’s barrier protective benefits in lung-endothelial cells (ECs) has not been clearly elucidated. Many vascular permeability-increasing agents weakened adherens junctions (AJ) integrity by inducing the reorganization of the actin cytoskeleton and stress fibers formation. Here, we hypothesized that metformin abrogated endothelial hyperpermeability and strengthen AJ integrity via inhibiting stress fibers formation through cofilin-1-PP2AC pathway.

**Methods:** We pretreated human lung microvascular ECs (human-lung-ECs) with metformin and then challenged with thrombin. To investigate the vascular protective effects of metformin, we studied changes in ECs barrier function using electric cell-substrate impedance sensing, levels of actin stress fibers formation and inflammatory cytokines IL-1β and IL-6 expression. To explore the downstream mechanism, we studied the Ser^3^-phosphorylation-cofilin-1 levels in scramble and PP2AC-siRNA depleted ECs in response to thrombin with and without metformin pretreatment.

**Results:** In-vitro analyses showed that metformin pretreatment attenuated thrombin-induced hyperpermeability, stress fibers formation, and the levels of inflammatory cytokines IL-6 and IL-β in human-lung-ECs. We found that metformin mitigated Ser^3^-phosphorylation mediated inhibition of cofilin-1 in response to thrombin. Furthermore, genetic deletion of PP2AC subunit significantly inhibited metformin efficacy to mitigate thrombin-induced Ser^3^-phosphorylation cofilin-1, AJ disruption and stress fibers formation. We further demonstrated that metformin increases PP2AC activity by upregulating PP2AC-Leu^309^ methylation in human-lung-ECs. We also found that the ectopic expression of PP2AC dampened thrombin-induced Ser^3^-phosphorylation-mediated inhibition of cofilin-1, stress fibers formation and endothelial hyperpermeability.

**Conclusion:** Together, these data reveal the unprecedented endothelial cofilin-1/PP2AC signaling axis downstream of metformin in protecting against lung vascular endothelial injury and inflammation. Therefore, pharmacologically enhancing endothelial PP2AC activity may lead to the development of novel therapeutic approaches for prevention of deleterious effects of ALI on vascular ECs.

## Introduction

The endothelium monolayer lines the luminal surface of the vascular tissue, forming a semipermeable barrier between circulating blood and underlying tissues, and regulating trafficking of blood cells and substrates across the vascular barrier (Claesson-Welsh et al., 2021). Endotheliopathy and endothelial hyperpermeability accompanied by pulmonary edema, influx of inflammatory cells into the lung is considered the hallmark of Acute Lung Injury (ALI), which progressively leads to Acute Respiratory Distress Syndrome (ARDS) (Bhattacharya and Matthay, 2013; Vassiliou et al., 2020). Additionally, the role of pulmonary EC dysfunction in the pathology of COVID-19 and other viral infections and their clinical sequelae has been corroborated (Ackermann et al., 2020; Jin et al., 2020; Mezoh and Crowther, 2021). Increase in circulating EC biomarkers have been reported to be associated with high disease severity and risk of death in COVID-19 patients (Osburn et al., 2022). Dysfunction of airway epithelial cell barrier that serves as pulmonary defense also leads to increased lung vascular permeability and edema (Finn et al., 2019). Although numerous studies have been conducted to find effective pharmacological therapy for ALI/ARDS, supportive mechanical ventilation remains the main current medical intervention to treat ALI/ARDS (Levitt and Matthay, 2012). However, the supportive mechanical ventilation is insufficient to reduce ALI/ARDS-associated mortality; as evident in COVID-19 infections. Therefore, delineation of molecular pathways regulating pulmonary endothelium barrier function may reveal novel therapeutic targets to potentially ameliorate lung inflammatory disease.

Metformin, a biguanide, is an effective glucose lowering drug widely prescribed for the management of type 2 diabetes (Aroda and Ratner, 2018). Beyond its anti-hyperglycemic effect, metformin exhibits anti-angiogenic, anti-inflammatory, and antioxidant properties (Joe et al., 2015; Kelly et al., 2015; Postler et al., 2021). Metformin has been shown to reduce the severity and mortality associated with sepsis and COVID-19 (Vaez et al., 2016; Ma et al., 2022). Many studies have investigated the protective effect of metformin in different models of lung injury including pulmonary hypertension, hyperoxia and ventilator-induced lung injury (Tsaknis et al., 2012; et al., 2015; Dean et al., 2016). In addition, metformin attenuates endotoxemia-induced pulmonary vascular injury by regulating endothelial barrier function suggesting a potential therapeutic role of metformin in ALI (Vaez et al., 2016).

The actin cytoskeleton rearrangement, increased stress fibers formation, and cell-cell retraction is an indispensable link between barrier function damage and increased permeability in vascular ECs under various pathophysiological conditions (Kasa et al., 2015; Dorard et al., 2019; Siddiqui et al., 2019). Cofilin-1 is a ubiquitously expressed actin-binding protein (ABP) that plays a pivotal role in actin filament dynamics (Hotulainen et al., 2005). Results from previous studies highlighted the critical role of cofilin-1 in modulating pulmonary capillary permeability (Gorovoy et al., 2008; Fazal et al., 2009). Ser^3^ dephosphorylation mediated activation of cofilin-1 facilitates its binding to actin, depolymerizes actin filaments, and reduces endothelial tension by disassembling stress fibers thus promote barrier function of lung microvascular endothelium (Fazal et al., 2009). The phosphorylation/dephosphorylation ratio of cofilin-1 is a critical determining factor for EC barrier function. Protein phosphatase 2A (PP2A) is a ubiquitously expressed, heterotrimeric, Serine/Threonine (S/T) phosphatase that accounts for a large fraction of phosphatase activity in eukaryotic cells. PP2A is composed of three subunits: a structural A, a catalytic C, and a regulatory B (Sangodkar et al., 2016). We and others previously showed that PP2A plays an essential role in modulating tissue inflammation by maintaining the phosphorylation/dephosphorylation balance of multiple proteins (Sun et al., 2015; Sontag et al., 2022). PP2A activators were shown to repress respiratory inflammation and vascular injury related to different pulmonary diseases (Peng et al., 2004; Rahman et al., 2016). In addition, PP2A is a direct upstream regulator of cofilin-1 dephosphorylation and its activation. Inhibition of PP2A by okadaic acid resulted in increased levels of phosphorylated cofilin-1 (Ambach et al., 2000; Oleinik et al., 2010; Tomasella et al., 2014). Based on these findings, we hypothesized that metformin-induced activation of PP2AC should inhibit cofilin-1 phosphorylation and subsequently activate cofilin-1 pathway to enhance endothelial barrier integrity.

In this study, we investigated the underlying mechanism(s) connecting metformin to the pulmonary endothelial barrier function and their role in response to metformin during endothelial injury. We show that the barrier protective effects of metformin occur via activation of PP2AC methylation, cofilin-1 dephosphorylation and counteracting aberrant stress fibers formation. Therefore, endothelial-specific upregulation of PP2AC activity could be a potential therapeutic target to reduce vascular leakage during many life-threatening lung diseases including ALI/ARDS.

## Materials and methods

### Reagents

Anti-PP2AC antibody (2038S), anti-phospho-LIMK1-Thr^508^/LIMK2-Thr^505^ (3841), and anti-phospho-AMPK-Thr^172^ (2531) antibodies were purchased from Cell Signaling Technology (Beverly, MA). Anti-PP2AC antibody (05-421) and metformin (1396309) was purchased from Millipore Sigma (Burlington, MA). Anti-actin (SC-477778) antibody was obtained from Santa Cruz Biotechnology (Santa Cruz, CA). GFP-PP2AC cDNA (cat. #RG201334) and mouse monoclonal turboGFP antibody, clone OTI2H8 (TA150041) was from OriGene (Rockville, MD). PP2A phosphatase assay kit was purchased from Upstate. Lipofectin-RNAiMAX transfection reagent (13778030), Protein G Dynabeads (1003D), Alexa Fluor 488 Phalloidin (A12379), Prolong Gold Antifade (P36941), and SYBR Green PCR master mix (4309195) were obtained from Invitrogen (Waltham, MA). FuGENE HD transfection reagent (E2311) was from Promega (Madison, WI). The enhanced chemiluminescence Western blotting detection reagents (RPN2106) were obtained from Thermo Fisher Scientific (Waltham, MA). Human α-Thrombin (HT 1002a) from Enzyme Research Laboratories (South Bend, IN). ECIS electrodes (8W10E + PET) were procured from Applied Biophysics (Troy, NY).

### Cell culture and cDNA transfection

Human lung microvascular ECs and EGM-2MV Bullet Kit (nos. CC-3156 and CC-4147) were obtained from Lonza (Walkersville, MD). ECs were cultured in EGM-2-MV medium in 0.2% gelatin-coated flasks and maintained at 37°C in a humidified atmosphere of 5% CO2 and 95% air. ECs between passages 3 and 7 were used for experiments. GFP-PP2AC cDNA was transfected in HLMVE cells using FuGENE HD transfection reagents. Briefly, 60%-70% confluent ECs were transfected in a 6-well using 1 μg GFP-PP2AC cDNA. After 48 h of transfection, ECs were used for different experiments.

### RNA interference

ECs at 50%–60% confluency were transfected with scramble-siRNA (sc-siRNA) and PP2AC-siRNA at a final concentration of 100 nM by using lipofectamine RNAi MAX according to the manufacturer’s instructions. Samples were collected at different time points to determine expression of target genes. After 48 h of transfection, ECs were used for different experiments. ON-TARGETplus SMARTpool-siRNA for human-PP2AC (Catalog ID:L-003598-01-0010) and ON-TARGETplus Non-targeting Control Pool (Catalog ID:L-001810-10-05) was obtained from Dharmacon.

### Transendothelial electrical resistance assay

Resistance across EC monolayers were measured by using an electrical cell substrate impedance-sensing system (ECIS; Applied Biophysics) as described previously (Siddiqui et al., 2019). ECs were seeded on gelatin coated standard array-gold plated electrodes. Cells were transfected with indicated siRNA or cDNA for 48 h. The change in TEER was measured across the monolayer in different experimental conditions and resistance was normalized to the initial value.

### Immunofluorescence

After 48 h of transfection, ECs were either left untreated or treated with 25 nM of thrombin in the presence and absence of metformin (10 mM for 2 h). Cells were fixed in 4% paraformaldehyde at room temperature for 10 min. After washing three times with PBS, cells were blocked with 1% BSA for 1 h at room temperature and then incubated with FITC-phalloidin (1:100 dilution) for 1 h at room temperature to stain actin stress fibers. Samples were mounted with prolong gold antifade reagent (Molecular Probes; Invitrogen) according to previous protocol (Siddiqui et al., 2019). Immunofluorescence microscopy was performed with a BZ-X710 all-in-one fluorescence microscope (Keyence, Itasca, IL). We randomly selected four to six fields per coverslip per experimental condition.

### Immunoprecipitation and Western blotting

For immunoprecipitation analysis, metformin treated ECs were washed with ice-cold PBS and immediately lysed in modified radioimmunoprecipitation assay (RIPA) buffer. Equal amounts of protein (500 μg) from each sample were precleaned with control IgG for 1 h at 4°C and then incubated with anti-PP2AC (5 μg) or anti-IgG (5 μg) antibodies overnight followed by the addition of protein-G Dynabeads for 2 h at 4°C to pull down the immunocomplexes. The samples were centrifuged at 1,000 × g for 2 min and the pellet containing beads were washed three times with RIPA buffer at 4°C. After centrifugation at 1,000 × g for 2 min, the beads were collected by removing supernatant buffer, and 30 μL of 2x Laemmli buffer was added to the beads and boiled. For Western blotting assay, equal amount of proteins were separated by SDS-PAGE and probed with different primary (1:1,000 dilution, overnight at 4°C) and the corresponding secondary antibodies (1:5,000 dilution, 1 h at room temperature). Densitometry analyses was performed using ImageJ software (NIH).

### Quantitative RT-PCR

Total cellular RNA was extracted from HLMVE cells using TRIzol reagent according to the manufacturer’s protocol. Reverse Transcription was performed with 1 μg of total RNA with a high-capacity cDNA reverse transcription kit from Applied Biosystems (Foster City, CA). Complementary DNA (cDNA) was then used as a template for amplification using the following primers: human IL-1β, forward, 5′-AGC​TAC​GAA​TCT​CCG​ACC​AC-3′ and reverse, 5′-CGT​TAT​CCC​ATG​TGT​CGA​AGA​A-3′; human IL-6, forward, 5′-CCT​GAA​CCT​TCC​AAA​GAT​GGC-3′ and reverse, 5′-TTC​ACC​AGG​CAA​GTC​TCC​TCA-3′; human β-actin, forward, 5′- AGC​CAT​GTA​CGT​TGC​TAT-3′ and reverse, 5′-GAT​GTC​CAC​GTC​ACA​CTT​CA-3′. The SYBR green gene expression assays were used for PCR amplification by using QuantStudio 6 Flex real-time PCR system from Applied Biosystems. Cycling conditions were as follows: 50°C for 2 min, 95°C for 10 min, followed by 40 cycles of 95°C for 15 s and 60°C for 1 min.

### Ser/Thr phosphatase assay

Immunoprecipitation based PP2A specific activity was analyzed by using Active PP2A DuoSet IC kit (R&D systems, MN) according to the manufacturer’s instruction. Equal amounts of cellular proteins were immunoprecipitated with an immobilized capture antibody specific for the catalytic subunit of PP2A. Ser/Thr phosphatase activity was determined by dephosphorylation of Ser/Thr phosphatase-specific phosphopeptide by active PP2A to generate free phosphate and unphosphorylated peptides. The inorganic phosphate released was detected by a sensitive dye-binding assay with malachite green and the activity of PP2A was calculated by determining the rate of phosphate released.

### Quantification and statistical analysis

GraphPad Prism 20.0 software was used for the statistical analysis. Normality distribution of data was checked using Shapiro–Wilk test. For continuous variables, two tailed Student’s t-test was performed to compare experiments containing two groups. One-way ANOVA with Tukey Post-Hoc performed for experiments having more than two groups. All the experiments were repeated three independent times and the data are presented as Mean ± S.D. All *p* < 0.05 values were statistically significant. ^∗∗∗^ denotes *p* < 0.001, ^∗∗^ denotes *p* < 0.01, ^∗^ denotes *p* < 0.05.

## Results

### Metformin attenuates thrombin-induced endothelial hyperpermeability and stress fibers formation

**FIGURE 1:**
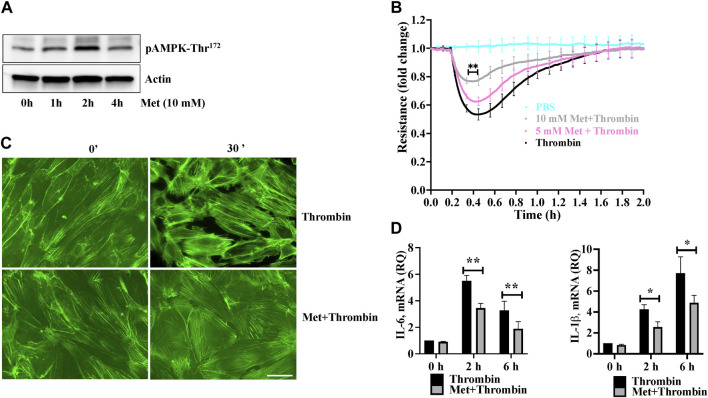
Metformin attenuates thrombin-induced endothelial hyperpermeability and stress fibers formation **(A)** Quiescent human lung-ECs were left untreated or treated with 10 mM of metformin for indicated time points. Equal amount of protein lysates from each condition were analyzed by Western blotting for phospho-AMPK-Thr^172^ and actin. Data was performed in three independent experiments **(B)** Quiescent human lung-ECs were analyzed for electrical cell impedance (represented as fold change index). ECs were treated with PBS, thrombin (25 nM) or pretreated with metformin (5 and 10 mM for 2 h) followed by thrombin challenge. Data analysis was performed in triplicate, and data are presented as mean ± SD; ^∗∗^ *p* < 0.01 vs. thrombin-treated group **(C)** Quiescent human lung-ECs were left untreated or treated with thrombin (25 nM) for indicated time points in the absence and presence of metformin (10 mM, 2 h). Representative immunofluorescence images of F-actin labelled with phalloidin-FITC (Scale bar: 20 μm). Data was performed in three independent experiments **(D)** qRT-PCR analysis of proinflammatory mediators. ECs were left untreated or treated with thrombin (25 nM) for indicated time points in the absence and presence of metformin (10 mM, 2 h). Data analysis was performed from three independent experiments, and are presented as mean ± SD; ^∗^ *p* < 0.05, ^∗∗^ *p* < 0.01 vs. thrombin-treated corresponding time group.

We first examined the effect of metformin on phosphorylation of AMPK at Threonine^172^. Human lung-ECs were pretreated with 10 mM of metformin for different time points. Figure 1A showed that metformin-induced highest upregulation of AMPK-phosphorylation-Thr^172^ residue at 2 h. Next, we studied the dose dependent effect of metformin on thrombin-induced endothelial hyperpermeability by measuring trans-endothelial electrical resistance (TEER). Human lung-ECs were pretreated with 5 and 10 mM of metformin for 2 h followed by stimulation with thrombin (25 nM). As shown in Figure 1B, thrombin challenge significantly decreased TEER values, an indicator of ECs barrier disruption and increased paracellular permeability. Intriguingly, metformin exhibited its pharmacological effects on ECs in a dose-dependent manner. The result showed that 10 mM metformin treatment most significantly protected against thrombin-induced ECs barrier disruption and increased paracellular permeability. Based on above results, we pre-treated ECs with 10 mM metformin for 2 h for further experiments. The role of F-actin stress fibers reorganization and ECs contraction in inflammation-induced paracellular gap formation is widely implicated. We next investigated the effect of metformin on thrombin-induced F-actin stress fibers formation. Our data showed a significant increase in stress fibers formation after 30 min of thrombin treatment, while pretreatment of ECs with metformin significantly decreased the level of stress fibers (Figure 1C). The upregulation of IL-6 and IL-1β is key marker of propagation of inflammation. Our qRT-PCR data showed a significant upregulation of thrombin-induced IL-6 and IL-1β mRNA levels while pretreatment of metformin significantly reduced IL-6 and IL-1β mRNA levels (Figure 1D), indicating anti-hyperpermeability and anti-inflammatory function of metformin in human lung-EC.

### Metformin attenuates thrombin-induced cofilin-1 phosphorylation

**FIGURE 2:**
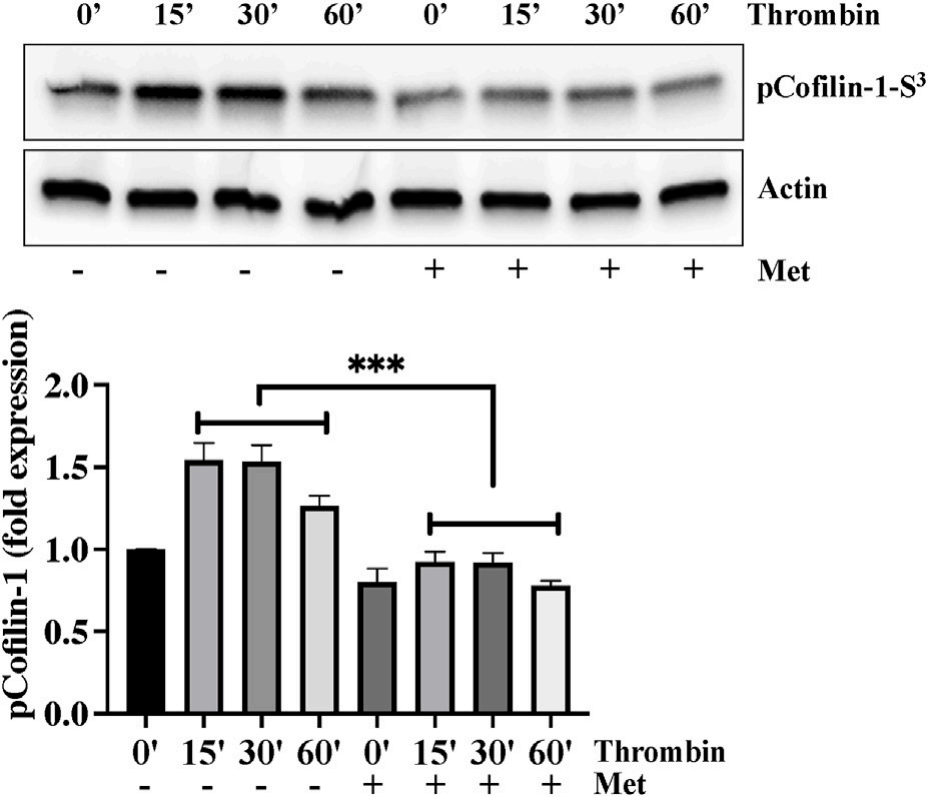
Metformin attenuates thrombin-induced cofilin-1 phosphorylation. Quiescent human lung-ECs were left untreated or treated with thrombin (25 nM) for indicated time points in the absence and presence of metformin (10 mM, 2 h) and equal amount of protein lysates from each condition were analyzed by Western blotting for phospho-cofilin-1-Ser^3^ and actin. The bar graphs represent quantitative analysis. Data analysis was performed from three independent experiments, and are presented as mean ± SD; ^∗∗∗^ *p* < 0.001 vs. thrombin-treated group.

We next investigated the importance of upstream regulation of stress fibers formation by metformin. Ser^3^ phosphorylation mediated deactivation of cofilin-1 plays an important role in actin cytoskeleton reorganization and stress fibers formation in response to inflammatory insult. We hypothesized that dephosphorylation of cofilin-1 could function as a positive regulator of barrier function in response to metformin. In Figure 2, Western blot analysis showed that thrombin induced cofilin-1 phosphorylation at its inhibitory Ser^3^ residue. This increased in cofilin-1 phosphorylation peaked at 15 min and began to decline after thrombin challenge. Interestingly, pretreatment of ECs with metformin significantly impaired thrombin-induced cofilin-1 phosphorylation at Ser^3^ demonstrating that cofilin activation is involved in reducing metformin mediated stress fibers formation.

### PP2AC silencing impairs metformin efficacy for cofilin-1 dephosphorylation to protect endothelial barrier function

**FIGURE 3:**
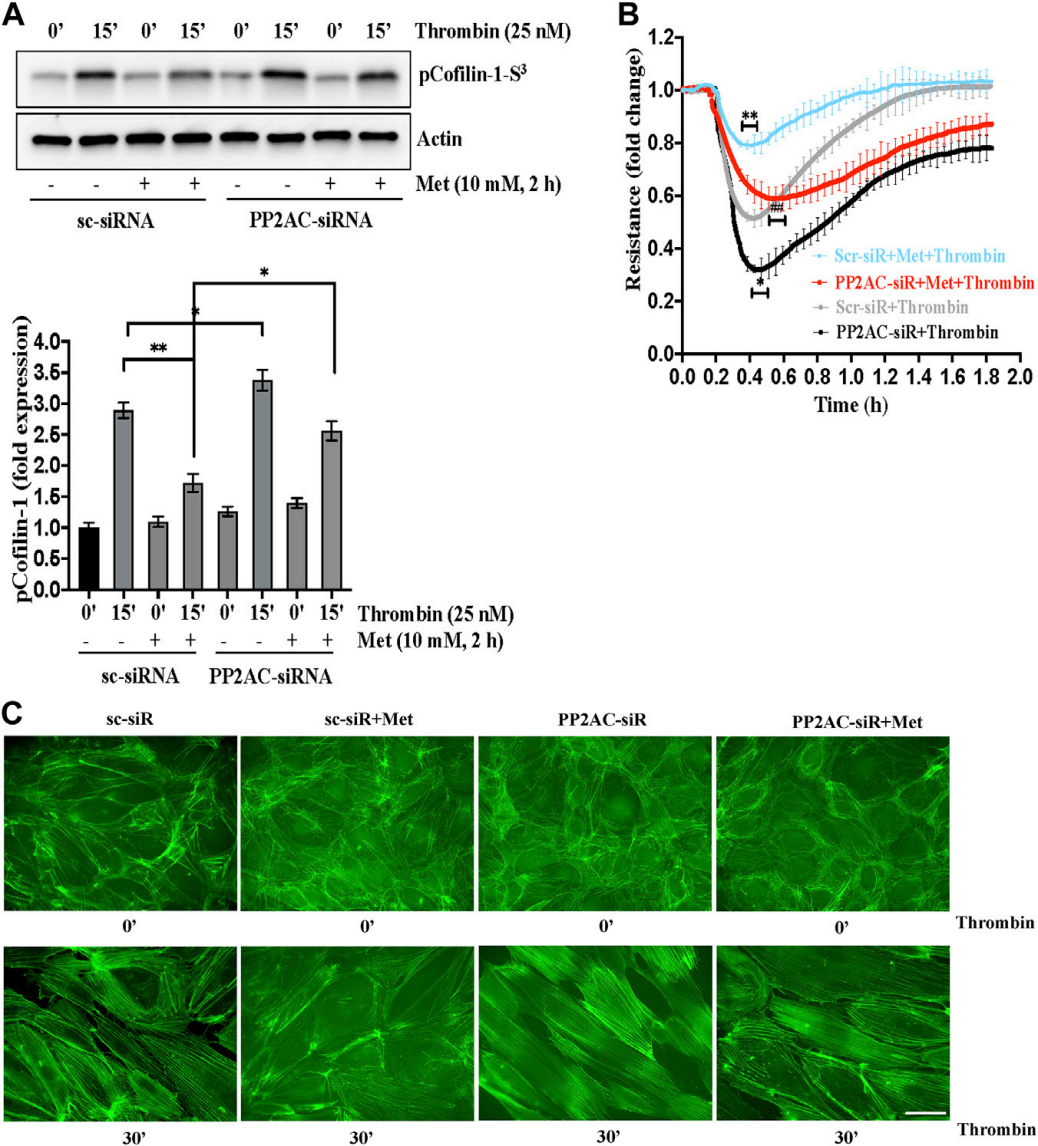
PP2AC silencing impairs metformin efficacy for cofilin-1 dephosphorylation to protect endothelial barrier function **(A)** Human lung-ECs were transfected with scramble-siRNA (sc-siRNA) or PP2AC-siRNA then left untreated or treated with thrombin (25 nM, 15 min) in the absence and presence of metformin (10 mM, 2 h). Equal amounts of proteins from each condition were analyzed by Western blotting for phospho-cofilin-1-Ser^3^ and actin. The bar graphs represent quantitative analysis. Data analysis was performed from three independent experiments, and are presented as mean ± SD; ^∗∗^ *p* < 0.001, ^∗^ *p* < 0.05 **(B)** Human lung-ECs were transfected with sc-siRNA or PP2AC-siRNA then left untreated or treated with thrombin (25 nM) in the absence and presence of metformin (10 mM, 2 h). Electrical cell impedance was analyzed (represented as fold change index). Data analysis was performed from three independent experiments, and are presented as mean ± SD; ^∗∗^ *p* < 0.001 vs. Scr-siRNA + thrombin treated group, ^∗^ *p* < 0.05 vs. Scr-siRNA + thrombin, ##*p* < 0.001 vs. Scr-siRNA + met + thrombin treated group **(C)** Human lung-ECs were transfected with sc-siRNA or PP2AC-siRNA then left untreated or treated with thrombin (25 nM, 30 min) in the absence and presence of metformin (10 mM, 2 h). Representative immunofluorescence images of F-actin labelled with phalloidin-FITC (Scale bar: 20 μm). Data was performed in three independent experiments.

PP2A family protein phosphatases are upstream regulator of cofilin-1 that are responsible for the dephosphorylation and reactivation of cofilin-1. Therefore, we determined whether metformin regulated cofilin dephosphorylation is caused by PP2AC. To this end, we knocked down PP2AC protein in ECs using siRNA approach and assessed phospho-cofilin-1 level. A strong downregulation of the PP2AC protein was observed at 48 h post-transfection of the PP2AC-targeting siRNA duplex (Supplementary Figure S1). As shown in Figure 3A, we found that metformin significantly inhibited thrombin-induced Ser^3^phosphorylation of cofilin-1 in sc-siRNA ECs, however, silencing of PP2AC resulted more significant increase in phospho-cofilin-1- Ser^3^ levels in response to thrombin. Interestingly, dephosphorylation of cofilin-1 by metformin was markedly abrogated in PP2AC silenced ECs after thrombin challenge. We next addressed whether silencing of PP2AC would dampen metformin ability to inhibit thrombin-mediated endothelial barrier dysfunction and stress fibers formation. Our results show that siRNA mediated deletion of PP2AC markedly augmented thrombin-induced endothelial barrier dysfunction and stress fibers formation as compared to sc-siRNA ECs (Figures 3B, C) and deletion of PP2AC significantly attenuated the efficacy of metformin in preventing thrombin-induced endothelial hyper-permeability (Figure 3B) and stress fibers formation (Figure 3C). Intriguingly, phospho-cofilin and TEER data showed that deletion of PP2AC has no effect at basal level, however, thrombin challenge caused much elevation of phospho-cofilin and barrier dysfunction in ECs with PP2AC deletion suggesting an essential role of PP2AC in metformin-mediated repressing ECs dysfunction in response to thrombin. Having demonstrated a key role for PP2AC in maintaining phospho-cofilin-1-Ser^3^ level and in mitigating thrombin induced ECs dysfunction, we speculated that metformin could be influencing PP2AC/cofilin-1 interaction. To address these possibilities, we first studied the interaction of cofilin-1 and PP2AC by co-immunoprecipitation assay. Metformin treated cell lysates were immunoprecipitated with anti-PP2AC antibody and immunoblotted for cofilin-1. Immunoblot analysis revealed PP2AC in the pull-down preparation, while metformin treatment did not induce the interaction of PP2AC with cofilin-1 (Supplementary Figure S2 **)**. To investigate whether the decrease in phospho-cofilin-1 level is a result of PP2AC phosphatase and not the lack of cofilin-1 associated kinase, we therefore sought to determine the effect of metformin on LIM kinase-1 phosphorylation. In accordance with the previous data, our immunoblot results showed that thrombin induced LIMK-1 phosphorylation at Thr^508^ residue, however this activated phosphorylation did not differ in response to thrombin ± metformin treatment (Supplementary Figure S3). Together, these results suggested a key role for PP2AC signaling as an upstream regulator of cofilin-1-Ser^3^ dephosphorylation in response to metformin in lung-ECs.

### Metformin induced PP2AC phosphatase activity

**FIGURE 4:**
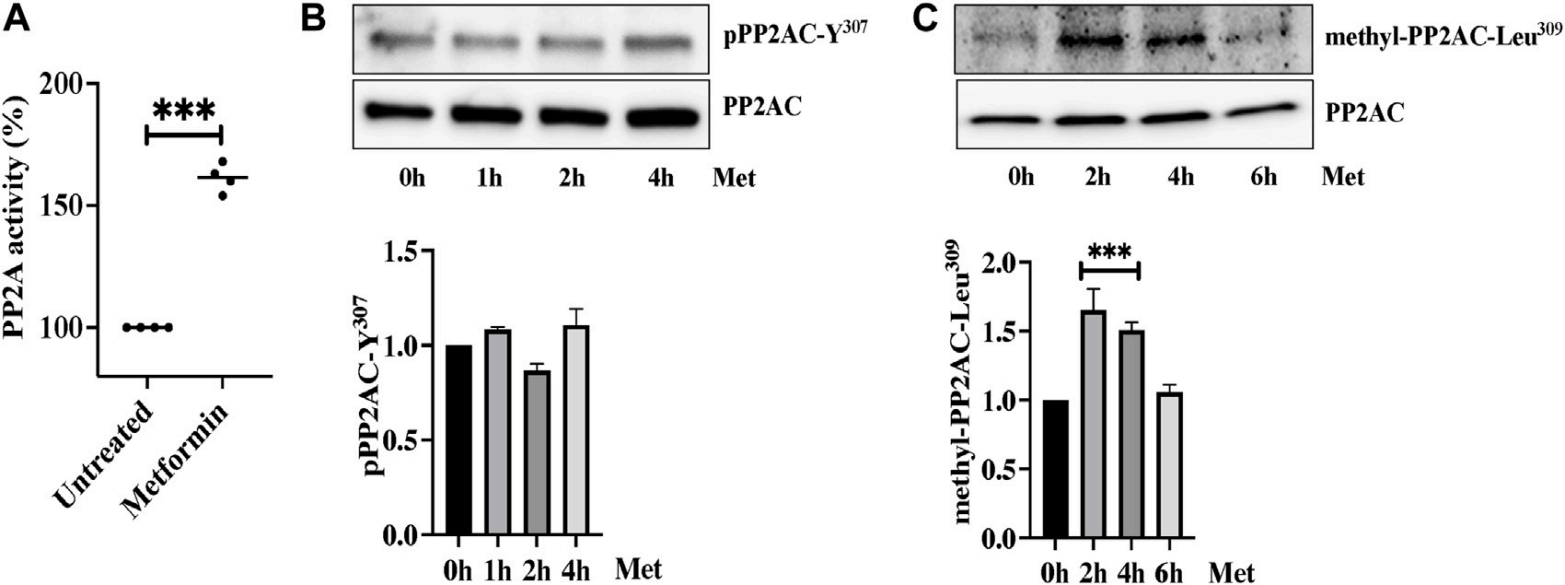
Metformin induced PP2AC phosphatase activity **(A)** Phosphatase activity was determined as described in the experimental procedure. Data analysis was performed from three independent experiments, and are presented as mean ± SD; ^∗∗∗^ *p <* 0.001 vs. untreated group. Quiescent human lung-ECs were treated with 10 mM of metformin for the indicated time points. Equal amounts of proteins from each condition were analyzed by Western blotting for **(B)** phospho-PP2AC-Y^307^ and PP2AC **(C)** methyl-PP2AC-Leu^309^ and PP2AC. The bar graphs represent quantitative analysis. Data analysis was performed from three independent experiments, and are presented as mean ± SD; ^∗∗∗^ *p* < 0.001 vs. 0 h treated group.

We next determined the effect of metformin on Ser/Thr phosphatase activity of PP2AC. We performed an immunoprecipitation based PP2A-specific activity assay and interestingly found significant increase in PP2A activity upon ECs stimulation with metformin (Figure 4A). We then sought to determine the exact mechanism by which metformin upregulates PP2AC activity. It is established that phosphorylation at Y^307^ and methylation at Leu^309^ are the two major modifications that have been shown to modulate Ser/Thr phosphatase activity of PP2AC. It is known that Y^307^ dephosphorylation of the catalytic subunit of PP2A leads to its functional activation while methylation at Leu^309^ regulates the biogenesis of the PP2A holoenzyme. In view of these observations, we first evaluated the effect of metformin on Y^307^ phosphorylation of PP2AC. Western blot analysis with PP2AC-Y^307^ specific-antibody revealed no significant difference in the level of Y^307^ phosphorylation of PP2AC after metformin treatment (Figure 4B). We subsequently evaluated Leu^309^ methylation of PP2AC which might be responsible for increased PP2AC activity in response to metformin. Metformin treated ECs lysates were immunoblotted with C-terminal leucine residue (Leu^309^) of the PP2A catalytic subunit specific antibody. Immunoblot results showed low levels of methylated PP2AC in untreated control, while metformin supplementation significantly upregulated methylation of PP2AC in ECs (Figure 4C).

#### Overexpression of PP2AC mitigated thrombin-induced hyperpermeability and cofilin-1 phosphorylation

**FIGURE 5:**
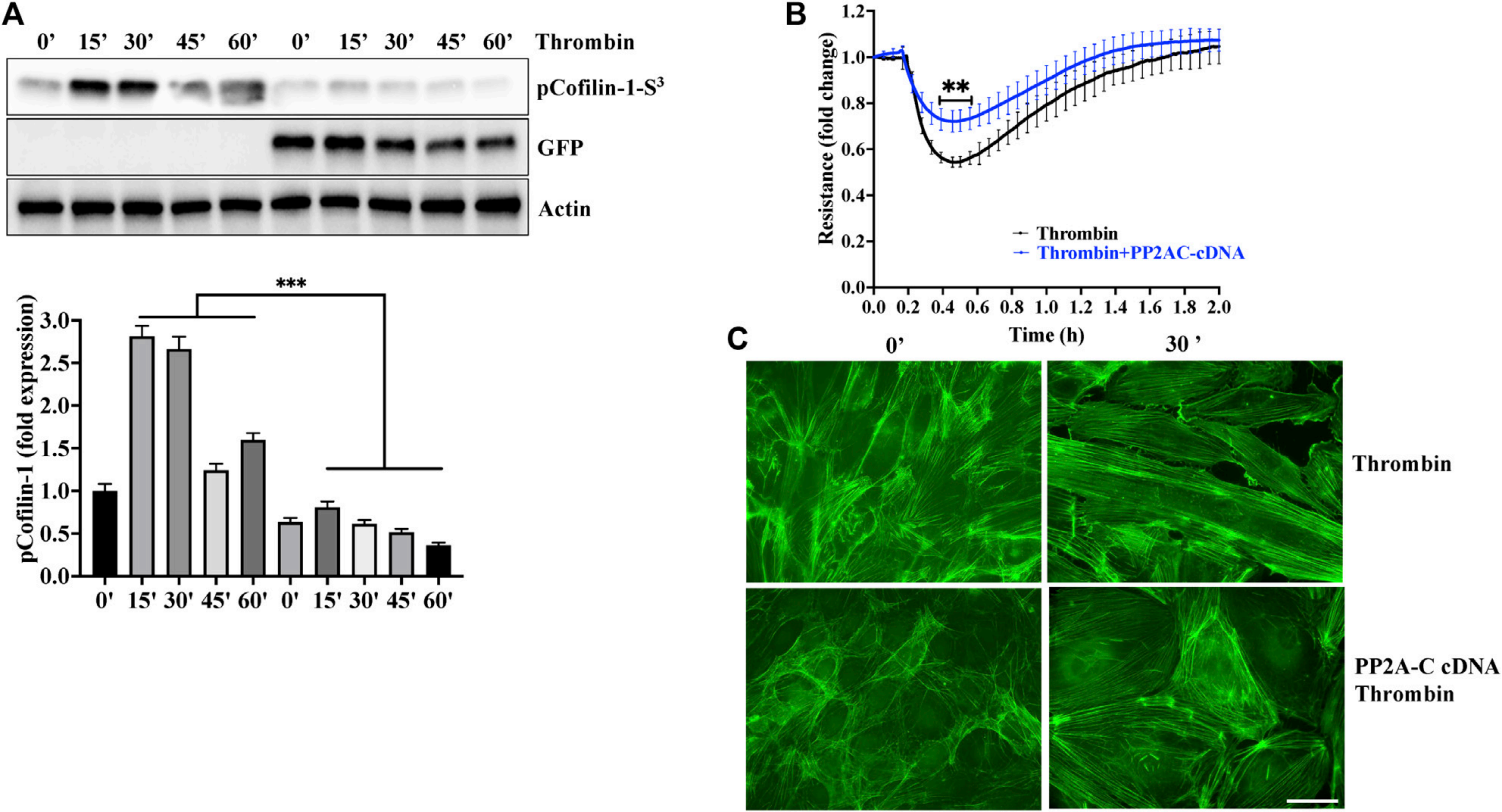
Overexpression of PP2AC mitigated thrombin-induced hyperpermeability and cofilin-1 phosphorylation. Human lung-ECs were transfected with PP2AC cDNA for 48 h and then **(A)** left untreated or treated with thrombin for indicated time points. Equal amount of proteins from each condition were analyzed by Western blotting for phospho-cofilin-1-Ser^3^, GFP and actin. The bar graphs represent quantitative analysis Data analysis was performed from three independent experiments, and are presented as mean ± SD; ^∗∗∗^ *p* < 0.001 vs. non-transfected thrombin-treated corresponding time point. Human lung-ECs were transfected with PP2AC cDNA for 48 h and then **(B)** left untreated or treated with thrombin (25 nM) and electrical cell impedance was analyzed (represented as fold change index). Data analysis was performed from three independent experiments, and are presented as mean ± SD; ^∗∗^ *p* < 0.01 vs. non-transfected thrombin-treated group **(C)** Left untreated or treated with thrombin (25 nM, 30 min) in the absence and presence of metformin (10 mM, 2 h). Representative immunofluorescence images of F-actin labelled with phalloidin-FITC (Scale bar: 20 μm). Data was performed in three independent experiments.

To gain further evidence for the role of PP2AC/cofilin-1 signaling in ECs barrier stability, we used gain of function approach by overexpressing PP2AC cDNA in lung-ECs. As downregulation of PP2AC abolished metformin ability to inhibit thrombin induced phosphorylation of cofilin-1 and endothelial hyperpermeability (Figure 3), we surmised that ectopic expression of PP2AC would suppress the ECs injury in response to thrombin. As expected, ectopic expression of PP2AC in human-lung-ECs significantly decreased cofilin-1 phosphorylation (increased cofilin-1 activation) (Figure 5A), endothelial hyperpermeability (Figure 5B) as well as actin-stress fibers formation (Figure 5C) in response to thrombin.

## Discussion

We demonstrated that metformin exhibited barrier protective effects against thrombin-induced hyperpermeability via restoring the balance between phosphorylated and non-phosphorylated cofilin-1 level in vascular ECs. Mechanistically, we show that metformin induced-methylation mediated activation of PP2AC. This increased in phosphatase activity of PP2AC inhibited S^3^ phosphorylation of cofilin-1 and limits phosphorylated cofilin-1-mediated stress fibers formation and subsequently mitigated endothelial hyperpermeability in response to thrombin.

Microvascular barrier hyperpermeability is a cardinal pathophysiological mechanism in lung inflammation that causes severe tissue injury and treatment with ECs barrier enhancing agents provide protection against ALI. Many studies in recent past have reported anti-inflammatory effects of metformin. Indeed, metformin treatment downregulates endotoxin-induced proinflammatory cytokines in vascular cells (Tian et al., 2019). Xian et al. observed that metformin treatment decreased NLRP3 inflammasome activation, IL-6 and IL-1β secretion, thereby attenuating LPS-induced ALI in mice (Xian et al., 2021). This study also highlighted the protective effect of metformin in SARS-CoV-2 induced pulmonary inflammation in hACE2 transgenic mice. Furthermore, metformin treatment alleviated paraquet-induced lung inflammation by promoting alveolar macrophages’ polarization towards M2 (Yuan et al., 2022). Furthermore, many studies have suggested that metformin exerts vascular protective effect on ECs. For instance, Arunachalam et al. showed that metformin mitigates hyperglycemia-induced endothelial senescence and apoptosis by regulating SIRT1 expression (Arunachalam et al., 2014). Another study showed that metformin dampened LPS-induced increased in permeability of rat lung-ECs (Jian et al., 2013). Many permeability-increasing agents such as thrombin promote actin stress fibers formation and lead to actinomyosin contraction are known to be induced ECs barrier dysfunction (Kasa et al., 2015; Yang et al., 2018; Siddiqui et al., 2019; Zhang et al., 2021; Zhao et al., 2021). Thus, it is tempting to investigate the effect of metformin on ECs activation and actin stress fibers formation, and interestingly we noticed that metformin antagonized thrombin effect on ECs barrier by inhibiting actin stress fibers formation. Our results are consistent with the aforementioned studies observed vascular protective properties of metformin.

There is strong evidence confirming that cofilin-1 is the central regulator of actin cytoskeleton dynamics and cofilin activation inversely corelates with endothelial barrier dysfunction. Cofilin-1 activation and its actin-depolymerization ability is abolished by cofilin-1 phosphorylation at S^3^ site (Hotulainen et al., 2005; Kiuchi et al., 2007). Indeed, transfection of phosphorylation-defective cofilin-1-S3A mutant abrogated thrombin-induced inflammation in lung-ECs (Fazal, Bijli et al., 2009). Recent study showed that fibrinogen inhibits stress fibers formation and enhanced endothelial barrier integrity by dephosphorylating cofilin-1 (Wu et al., 2021). Here, we found that metformin effectively decreased phospho-cofilin-1-Ser^3^ level, which demonstrates that cofilin-1 mediated inhibition of stress fibers formation could be one of the mechanisms downstream of metformin to antagonize detrimental effect of thrombin on ECs barrier integrity. Furthermore, the level of cofilin-1-S^3^ and its activity depend on phosphorylation by kinases and dephosphorylation by specific phosphatase. Numerous studies indicate PP2A isoforms as cofilin-specific phosphatases (Ambach et al., 2000; Zhan et al., 2003; Wolterhoff et al., 2020) and interestingly PP2A has key functional role in regulation of actin dynamics (Wolterhoff et al., 2020). Our data show that PP2AC is required for metformin-dependent inhibition of cofilin phosphorylation to preserve AJs integrity in lung-ECs. Although it has been reported that PP2A interact with cofilin-1 to induced its dephosphorylation in lung carcinoma cell line A549 (Oleinik et al., 2010), we were unable to demonstrate any direct cofilin-1-PP2AC interaction, suggesting that the interaction in ECs may be weak or indirect or this interaction could be cell type dependent. Another important prospective about PP2AC is that it constitutively bound to AJs proteins and maintains ECs barrier function. Interestingly, a previous study showed that Semaphorins 3A, known to regulates tumor angiogenesis, downregulated PP2A activity that leads to S^665^ phosphorylation of VE-cadherin and subsequently destabilized cell-cell junction in human-brain-ECs (Le Guelte et al., 2012). In contrast to this result with human-brain-ECs where PP2AC knockdown induced barrier disruption basally, no decreased in human-lung-ECs barrier function on PP2AC knockdown was seen in the current study. Thus, PP2AC could work at two different but equally important pathways that include AJs protein and actin cytoskeleton dynamic to maintain ECs physiological function. Thus, in the future it will be interesting to study whether metformin could regulate the phosphorylation level of junctional proteins through PP2AC to regulate endothelial ECs barrier function, and vascular regulation. Nonetheless, we found that PP2AC activity is essential for barrier protective function of metformin. Further studies are warranted to determine the lung protective effect of metformin in *in vivo* studies using PP2AC knockout mice. Our results indicate that overexpression of PP2AC attenuates thrombin-induced cofilin-1-Ser^3^ phosphorylation, stress fibers formation and ECs barrier dysfunction. These data were consistent with those of a previous study showing that FTY 720 mediated activation of PP2A significantly decreases microvascular permeability and respiratory inflammation (Peng et al., 2004; Rahman et al., 2016).

The activity and substrate specificity of PP2A are regulated by protein-protein interactions and posttranslational modifications. Phosphorylation on Y^307^ within PP2AC was identified as a key negative regulator of PP2A while LCMT-1/PME-1 mediated methylation of the C-terminal leucine^309^ residue has been reported to be important for its activation (Chen et al., 1992; Tolstykh et al., 2000). We observed that increased PP2AC methylation upon EC stimulation with metformin could intimately linked with cofilin-1 activation and actin cytoskeleton dynamics. This hypothesis is further supported by the strong functional link between cytoskeletal reorganization and PP2AC methylation. Noticeably, PP2A methylation is essential for Fyn-dependent neuritogenesis and inhibiting PP2A methylation interferes with deregulation of F-actin dynamics in N2a cells (Sontag et al., 2022). However, due to the intricacy of PP2A methylation regulation by LCMT-1 and PME-1, further comprehensive studies will be needed to fully interpret the underlying mechanisms regulating metformin-mediated activation of PP2A.

Collectively, our results provide molecular insights into the vascular protective actions of metformin. We demonstrated that thrombin-induced phospho-cofilin-1-Ser^3^ increased stress fibers level, and concomitantly ECs dysfunction. The pretreatment of metformin attenuated the deleterious effect of thrombin on AJs via PP2AC-dependent molecular mechanism. Our data suggest the efforts to improve PP2AC activity will provide new avenues of research to find potential therapeutic drugs for counteracting EC dysfunction-associated lung injury.

## Data availability statement

The original contributions presented in the study are included in the article/Supplementary Material, further inquiries can be directed to the corresponding author.

## Author contributions

MRS and TS conceived and designed the study. MRS and NM performed the experiments and analyzed the data. MRS and TS draft the manuscript. HF and MRS critically revised the manuscript. All authors contributed to the article and approved the submitted version.
