# Supplementary figures and images for: Metformin alleviates lung-endothelial hyperpermeability by regulating cofilin-1/PP2AC pathway
Source: Front Pharmacol. 2023 Jun 8;14:1211460. doi: 10.3389/fphar.2023.1211460 (PMC10285707; doi:10.3389/fphar.2023.1211460)

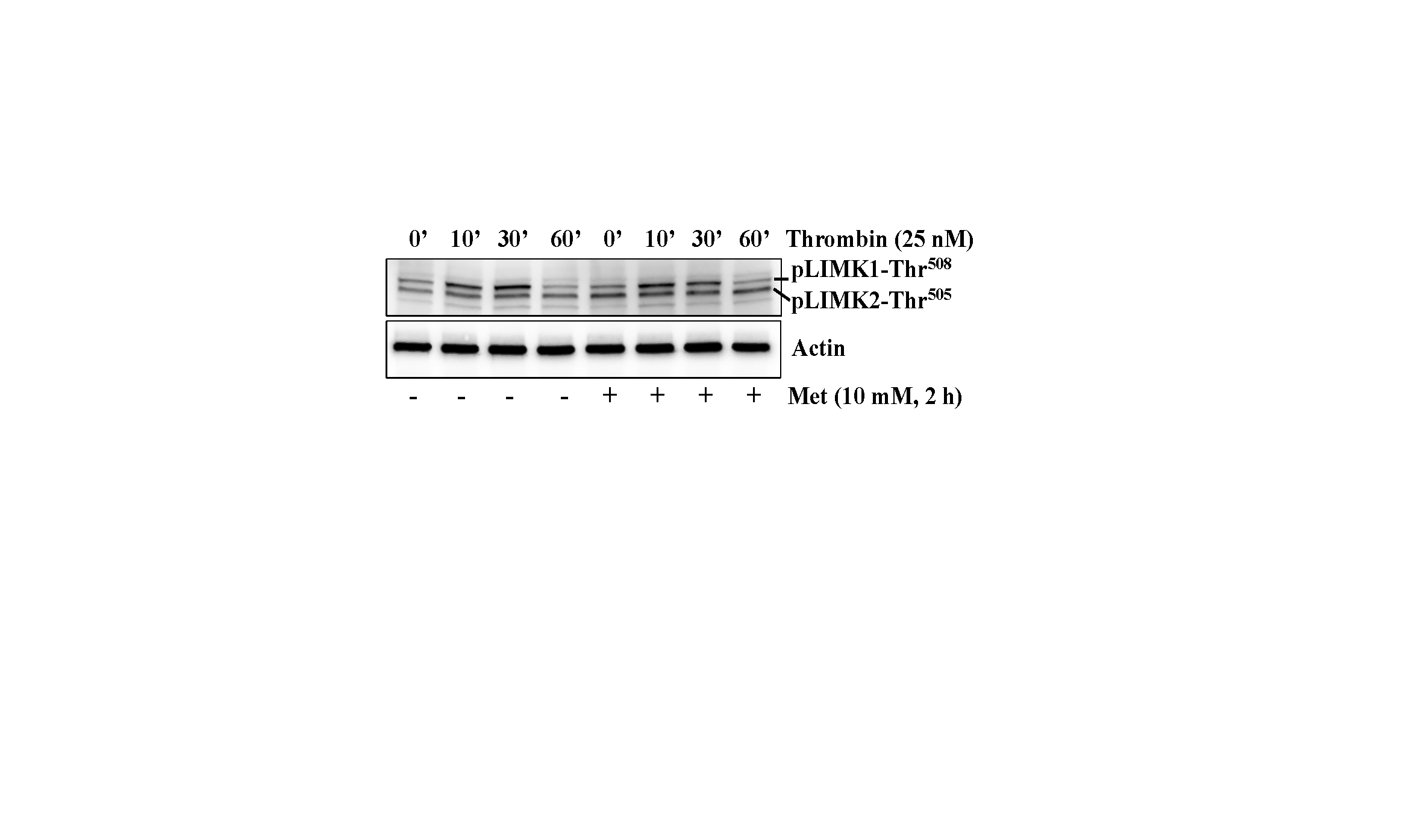

Supplement: Supplementary file 1 [file Image3.TIFF]

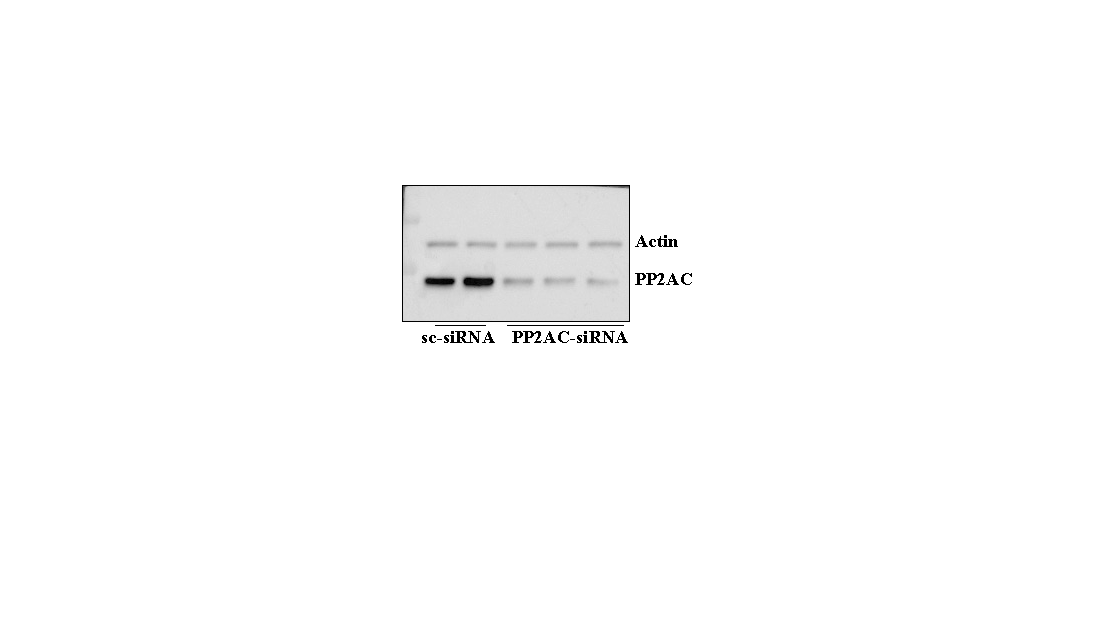

Supplement: Supplementary file 2 [file Image1.TIFF]

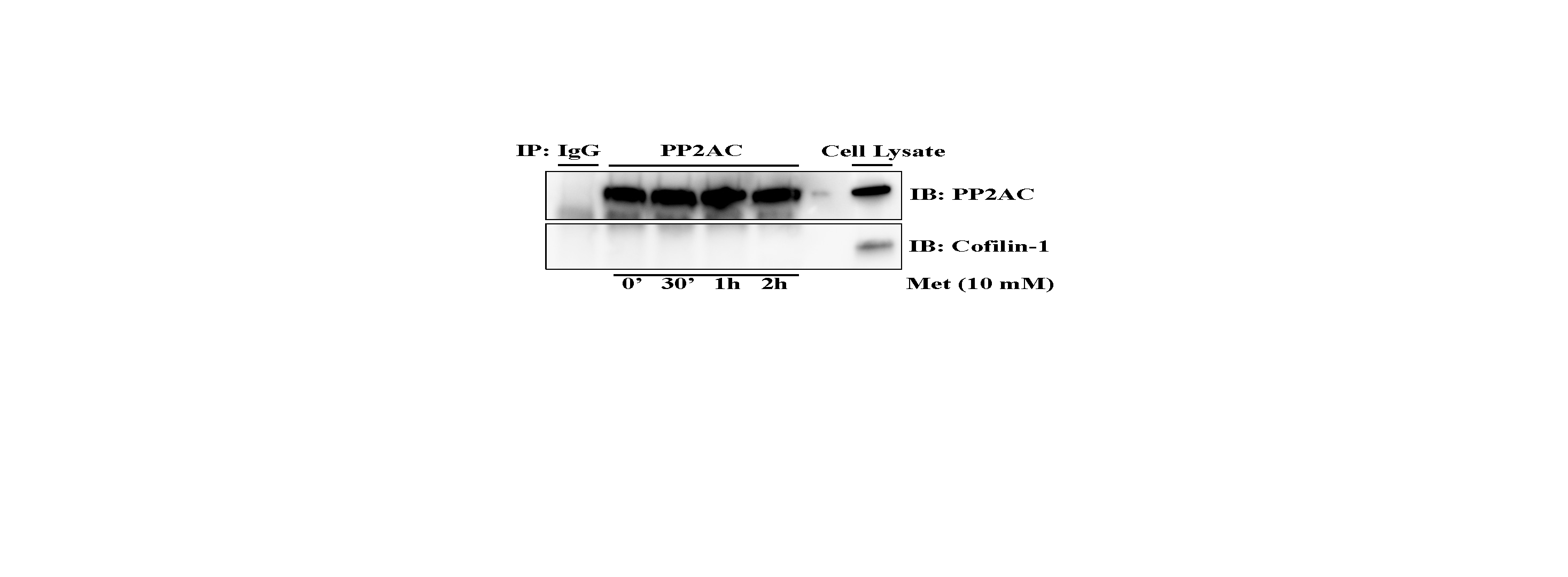

Supplement: Supplementary file 3 [file Image2.TIFF]
